# A Method of Filling Root-Canals

**Published:** 1895-01

**Authors:** James H. Daly

**Affiliations:** Boston


### A METHOD OF FILLING ROOT-CANALS.[1]

BY DR. JAMES H. DALY, BOSTON.

The few thoughts I have to present to you this evening were suggested, first, by a case of necrosis caused by the slovenly application of arsenic; second, by an article in the *Dental Cosmos* of last year, by Nelson S. Shields, stating that gold-foil was the only material for a root-filling.

The application of an arsenous acid dressing that a pulp may be destroyed is an all-important feature in the final result as to whether the filling of a root-canal is to be successful or not.

If the merest particle of the acid is allowed to escape from the cavity and come in contact with gum-tissue, then commences a destruction of it which is minor in importance to what follows, —destruction of the septum of bone between the teeth. An inflammation is here established in the pericementum that is often thought to be caused by the pulp, and unnecessarily long and protracted treatment of the pulp-canal is the result. It is of the utmost importance that no part of the arsenical dressing come in contact with the tissue surrounding the tooth. A very severe case

---

[1] Read before the American Academy of Dental Science, June 6, 1894.

of necrosis came under my observation, caused by an unnecessarily large amount of acid being placed in the cavity in the first place, allowed to remain for a very long time, and the dressing so placed that there was a constant escape from the cavity of a small amount of the arsenous acid. The result was that a large area of bone was destroyed, necessitating removal.

In destroying the dental pulp, a positive method, and one not followed by unpleasant features, is to administer gas and forcibly remove the pulp with a barbed broach. That patients are not always willing to have this done we are all aware.

In filling root-canals, I assume that we agree that the important feature is to thoroughly seal the apex of the root, not to force material through, neither to allow a small space to remain unfilled. A few operators, and I speak advisedly when I say a few, *can fill* tortuous canals, and have them hermetically sealed with gold-foil; but the majority are unable to successfully do so, nor is it necessary to thus exhaust both patient and operator. For that reason, I find that a gold wire tapered at the end may be made to perfectly fill the end of the canal.

With suitable-sized gold wire for the case at hand, taper the wire and sharpen the extreme point, that it may easily be forced through the end of the canal,—not sufficiently to seriously wound the tissues, but just enough to prick a little, the patient surely letting us know when that point is reached. Now mark on the gold-wire broach the exact length of the canal, together with the portion of the wire through the end, and remove and file off the tiny sharpened portion; again insert in the canal and force to the end of the root, to see that there is no pain upon reinsertion; again remove and file into and almost through the wire one-sixteenth of an inch from the end; then again insert, and, having forced to the end of the canal, twist the gold broach slightly, leaving the small portion that was nearly filed off at the end of the root, thus securely sealing it with a "royal" metal.

The remainder of the canal may be filled with any of the root materials,—gutta-percha, oxychloride, or zinc phosphate, as the operator prefers. It is true that it requires patience and care-taking to successfully cope with some of the flat canals found in superior bicuspids, also in mesial roots of lower molars, or large open foraminæ in the teeth of children; but if a royal metal is desired, it has seemed to me that this was an expeditious and comparatively easy way to obtain it.

